# Modelling of Process-Induced Deformation for Composite Parts Considering Tool-Part Interaction

**DOI:** 10.3390/ma13204503

**Published:** 2020-10-11

**Authors:** Wei Qiao, Weixing Yao

**Affiliations:** 1 Key Laboratory of Fundamental Science for National Defense-Advanced Design Technology of Flight Vehicle, Nanjing University of Aeronautics and Astronautics, Nanjing 210016, China; m18168071852@163.com; 2 State Key Laboratory of Mechanics and Control of Mechanical Structures, Nanjing University of Aeronautics and Astronautics, Nanjing 210016, China

**Keywords:** fiber-reinforced polymer-matrix composites, finite element analysis, path-dependent model, process-induced deformation, tool-part interaction

## Abstract

Residual stresses are generated by tool-part interaction due to the large difference in the coefficients of thermal expansion (CTE) between the tool and the composite part, resulting in more process-induced part deformation. In this paper, a 3-D numerical model considering the influence of tool-part interaction is proposed to predict the deformation in complex-shape composite parts. In this numerical model, the existing path-dependent model is improved to consider the effect of tool-part interaction by adding the residual stress generated by tool-part interaction, and a simplified self-consistent micromechanics model is selected to predict the composite mechanical properties in the viscous and rubbery stages. The predicted and experimental spring-in angles of L- and U-shaped parts are compared. A good agreement shows the validity of the proposed numerical model. A parametric study is performed and the influence of part structural parameters on the spring-in angle is analyzed quantitatively. The results show that the spring-in angles caused by chemical shrinkage and tool-part interaction decrease with the increase of part thickness, but that caused by thermal contraction is almost constant.

## 1. Introduction

Benefiting from higher strength and stiffness, advanced fiber-reinforced composites have been widely applied in many areas such as aerospace and ship building. Due to thermal anisotropy of composite materials as well as resin chemical shrinkage and tool-part interaction, residual stresses are generated in composite parts during curing [1,2]. After curing, these residual stresses will cause the geometrical deformation of composite parts that are removed from the tool [3,4,5]. It is very difficult to meet the manufacturing technical requirements when assembling deformed parts. Therefore, it is important to accurately predict the deformation of composite parts so as to improve the assembly quality and reduce the manufacturing cost.

In the past decades, many numerical models have been developed to predict process-induced deformation of composite parts. In order to accurately describe the change of composite mechanical properties during curing, different constitutive models were proposed in numerical models. These constitutive models can be simply divided into three types: elastic [6,7], viscoelastic [8,9,10,11,12,13] and simplified viscoelastic models [14,15,16,17,18]. The cure hardening instantaneously elastic constitutive model proposed by Bogetti et al. [6] is a typical linear elastic model, which assumes that the resin modulus increases linearly with the cure degree. However, experimental investigation has shown that resin exhibits viscoelastic behavior under high temperature, and the elastic model has some contradictions with experiment results [19]. A viscoelastic model proposed by Zobeiry et al. [8] can more accurately describe the viscoelastic mechanical behavior of resin-dominated composites, but it requires extensive material data, a lot of running time and huge storage space for computation. In order to reduce the dependence on experimental results, Svanberg et al. [14,15] proposed a path-dependent model, which assumes that the resin modulus is completely relaxed in the rubbery state and no relaxation occurs in the glassy state. According to the conclusion of Ding et al. [20], there is insignificant difference in the calculated deformation of curved parts with path-dependent and viscoelastic models. It can therefore be concluded that although the path-dependent model has greatly simplified the composite properties during curing, it captures the main mechanism that causes deformation. The path-dependent model is considered to be a popular constitutive model, which has been successfully applied to the deformation prediction of L-shaped laminates [21], curved laminates [18] and curved C-shaped spar [22]. However, the path-dependent model is mainly used to calculate the spring-in deformation caused by thermal contraction and chemical shrinkage without considering the influence of tool-part interaction.

The main factor that causes tool-part interaction is the large difference in the coefficients of thermal expansion (CTE) between the tool and the composite part [23,24]. Due to the tool-part interaction, some asymmetric residual stresses in the thickness direction are caused during the heating process, which further cause bending deformation of parts after demolding [25,26,27]. Two analytical models were proposed respectively by Arafath et al. [25] and Twigg et al. [26] to throw insight into the physical mechanism of warping deformation in flat laminates caused by the tool-part interaction. In numerical models, an interfacial sliding friction [27,28,29] or a cure hardening elastic shear layer [25,30,31] is introduced between the tool and the part to simulate the effect of tool-part interaction. Most previous investigations on the tool-part interaction mainly focus on the flat parts, while the research on complex shape parts considering tool-part interaction is relatively less.

The main objective of this paper is to extend the application of the path-dependent model so that it can consider the influence of tool-part interaction. A 3-D numerical model based on a modified path-dependent model to predict the process-induced deformation for complex-shape composite parts is realized considering the effect of tool-part interaction. The existing path-dependent model is improved to consider the effect of tool-part interaction by adding the residual stress generated by tool-part interaction. A simplified self-consistent micromechanics model is used to predict composite mechanical properties in the viscous and rubbery states. The predicted spring-in angles of L- and U-shaped parts with different thicknesses and lay-ups are compared with experimental data. Finally, a parametric study is performed to analyze quantitatively the influence of structural parameters on spring-in angles based on the proposed numerical model.

## 2. Theoretical Models

### 2.1. Thermo-Chemical Model

The thermo-chemical model for anisotropic composite materials includes the heat transfer model, internal heat model and the reaction kinetic equations. The governing equation of the thermo-chemical model can be expressed as follows [14]:

(1) $$k_{x}\frac{\partial^{2}T}{\partial x^{2}}+k_{y}\frac{\partial^{2}T}{\partial y^{2}}+k_{z}\frac{\partial^{2}T}{\partial z^{2}}+Q=\rho C\frac{\partial T}{\partial t}$$

where *k_x_*, *k_y_* and *k_z_* are thermal conductivity coefficients of the composite; *ρ* and *C* are the density and the specific heat capacity coefficient of the composite, respectively; *T* is the transient temperature; *Q* is the internal heat generated by the chemical reaction of the resin, which can be determined as follows [14]:

(2) $$Q=\rho_{r}(1-V_{f})H_{r}\frac{d\alpha }{dt}$$

where *ρ_r_* is the resin density; *V_f_* is the fiber volume fraction; *H_r_* is the total amount of heat generated by the resin during curing; *α* is the cure degree; *dα/dt* is the cure rate, and the cure kinetic equation of 3900-2 resin is defined as [32]:

(3) $$\frac{d\alpha }{dt}=A\exp(-\frac{\Delta E}{RT})\alpha^{m}{\left(1-\alpha \right)}^{n}$$

where the gas constant *R* is 8.314 J/mol·K; *A* and Δ*E* are the pre-exponential coefficient and the activation energy, respectively; *m* and *n* are the exponential constants. The thermal properties of T800HB/3900-2 composite are taken from the Toray Company and are given in Table 1. The cure kinetic constants of 3900-2 resin are listed in Table 2.

### 2.2. Improved Path-Dependent Model

**Figure 1:**
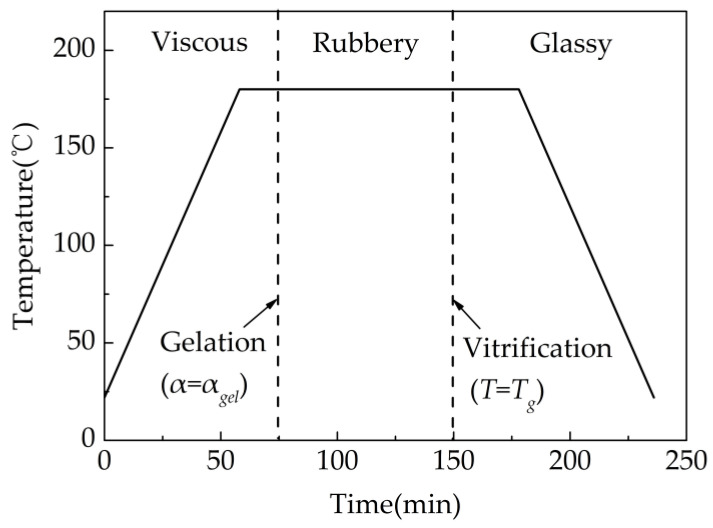
Various stages of composite properties during curing.

According to the development of resin mechanical properties during curing, the curing process was divided into three stages, including viscous, rubbery and glassy stages, with gelation and vitrification points forming the boundary, as shown in Figure 1. In the path-dependent model, the composite mechanical properties are assumed constant within each stage, and the transition from the rubbery stage to the glassy stage presents a step-like change. The stress increment equation of this model can be expressed as [14]:

(4) $$\Delta \sigma_{i}=\left\{ \begin{array}{ll} C_{ij}^{r}\Delta \varepsilon_{j}^{eff}-S_{i}(t) & T\geq T_{g}\\ C_{ij}^{g}\Delta \varepsilon_{j}^{eff} & T<T_{g} \end{array}\right.$$

where *S_i_* is the history state variable and can be given by [14]:

(5) $$S_{i}(t+\Delta t)=\left\{ \begin{array}{ll} 0 & T\geq T_{g}\\ S_{i}(t)+(C_{ij}^{g}-C_{ij}^{r})\Delta \varepsilon_{j}^{eff} & T<T_{g} \end{array}\right.$$

where $C_{ij}^{r}$ and $C_{ij}^{g}$ are the constant stiffness matrices for the composite in the rubbery and glassy stages, respectively. *T_g_* is the glass transition temperature. *σ_i_* and $\Delta \varepsilon_{j}^{eff}$ denote the stress tensor and strain tensor, respectively.

An important assumption for the existing path-dependent model is that no residual stress is preserved in the viscous stage. Because the resin has a strong fluidity prior to the gelation, the thermal contraction and chemical shrinkage of the resin are difficult to generate residual stress. However, it was reported in experiments [24] that prior to the gelation, an interaction between the fiber bed and the tool was noticed, and it indicated that residual stress existed in fibers. In this paper, the residual stress generated by the tool-part interaction in the viscous stage was calculated. Then, the stress increment equation of a new path-dependent constitutive model can be determined as follows:

(6) $$\Delta \sigma_{i}=\left\{ \begin{array}{ll} C_{ij}^{v}\Delta \varepsilon_{j}^{eff} & \alpha <\alpha_{gel}\\ C_{ij}^{r}\Delta \varepsilon_{j}^{eff}-S_{i}(t) & \alpha \geq \alpha_{gel}\;\mathrm{and}\;T\geq T_{g}\\ C_{ij}^{g}\Delta \varepsilon_{j}^{eff} & \alpha \geq \alpha_{gel}\;\mathrm{and}\;T<T_{g} \end{array}\right.$$

where *α_gel_* is the cure degree at the gelation point, and its value is 0.518 for 3900-2 resin [32]. *T_g_* for T800HB/3900-2 composite can be approximately expressed as:

(7) $$T_{g}=2.67+11.2\alpha +168.3\alpha^{2}$$

### 2.3. Simplified Self-Consistent Micromechanics (SCFM) Model

The mechanical properties of composites in the viscous and rubbery stages cannot be obtained by experiments except for the glassy mechanical properties, and are usually predicted by some analytical models [21]. In these analytical models, the SCFM model is considered to be relatively reliable with respect to the prediction of composite mechanical properties [21,33]. However, the existing SCFM model requires too much material property data of the fiber and the resin. In this paper, great efforts are made to simplify this model.

The composite modulus parallel to the fiber direction are dominated by the fiber modulus, so it is assumed that the longitudinal modulus *E*_11_ of the composite is constant during curing. In the viscous and rubbery stages, the resin is generally considered to be an incompressible material, so the resin Poisson’s ratio *v_r_* is 0.5, and the transverse Poisson’s ratio *v*_23_ of the composite is assumed to be 1.0, which have been experimentally confirmed by Ersoy et al. [33]. However, in order to prevent the generation of non-positively determined material matrix, *v*_23_ is determined as 0.995 in the numerical model. Moreover, the resin modulus in the viscous and rubbery states is much smaller than the fiber modulus. Therefore, a simplified SCFM model can be derived as follows:

(8) $$\left\{ \begin{array}{l} \nu_{23}=0.995\\ G_{12}=G_{13}=G_{23}\;=\frac{1+V_{f}}{3(1-V_{f})}E_{r}\\ E_{2}=4G_{12}\; \end{array}\right.$$

In this paper, the simplified SCFM model is selected to predict the mechanical properties of the composite in the viscous and rubbery stages. The input parameters of this model are reduced from 9 to 2, reducing the dependence on the fiber property and the resin property.

## 3. Experiment and Numerical Modeling

### 3.1. Material and Configuration

**Figure 2:**
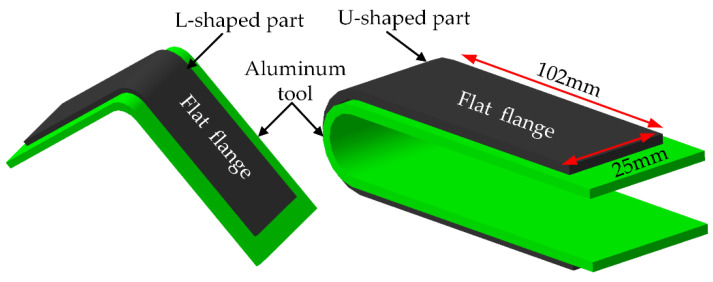
Geometry and shape of the specimens.

The process-induced deformation of some L- and U-shaped composite parts manufactured of T800HB/3900-2 prepreg (Toray) were experimentally investigated in literature [34]. The prepregs were placed on the external surface of the aluminum (6061-T6) tool and were cured in the autoclave successively. After curing, all cured specimens were trimmed to a flat flange with a length of 102 mm, a width of 25 mm, as shown in Figure 2. The average thickness of one layer was 0.16 mm, and the average fiber volume fraction was 62.5%. The specific values for thermoelastic properties of T800HB/3900-2 composite in the glassy state are listed in Table 3. In this paper, the thickness of the aluminum tool and the shear layer are respectively 4 mm and 0.2 mm and their thermoelastic properties are shown in Table 4. According to the manufacturer’s recommended cure cycle (MRCC), the temperature holds 180 °C for 2 h, the heating and cooling rates are respectively 2.7 °C/min and −2.7 °C/min, and the vacuum and autoclave pressures are respectively 0.16 MPa and 0.6 MPa.

The thermoelastic properties of 3900-2 resin are shown in Table 5. The measured glassy modulus (fully cured) of 3900-2 resin is 4710 MPa [25]. The viscous modulus of the resin at low cure degree is estimated to be 0.0471 MPa [25]. The rubbery modulus of the resin is assumed to be 1% the glassy modulus, according to the research of Svanberg et al. [14]. Finally, the required mechanical properties of T800HB/3900-2 composite in viscous and rubbery states will be calculated using Equation (8).

### 3.2. Numerical Implementation

**Figure 3:**
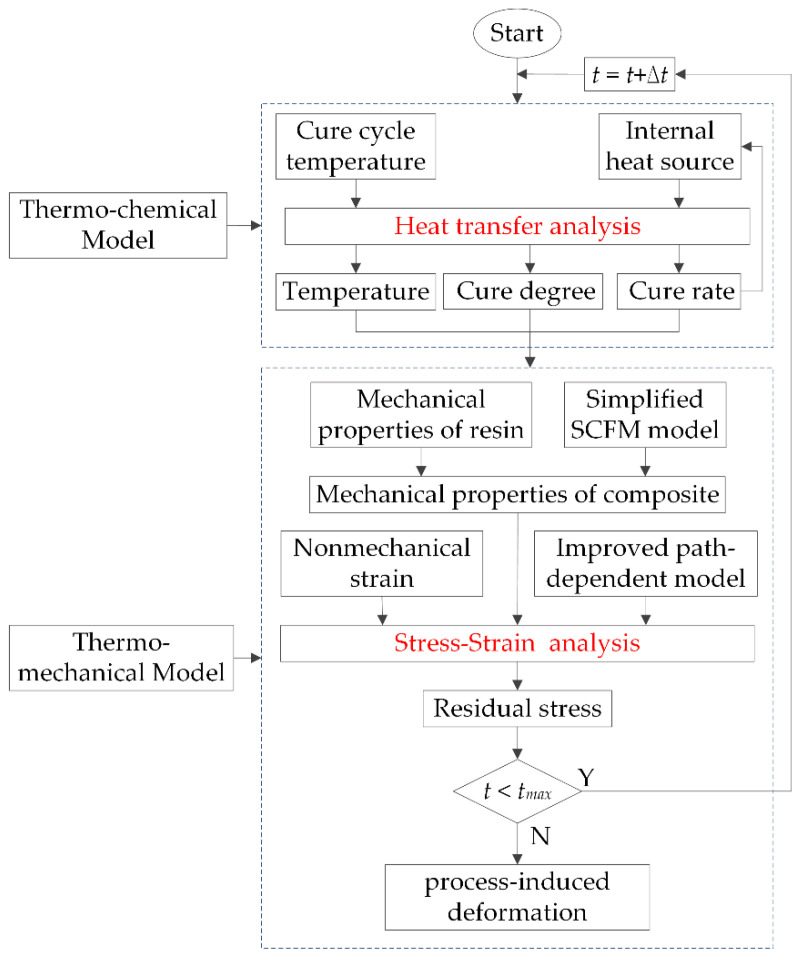
Simulation calculation process of numerical model.

A 3-D numerical analysis model based on the commercial software ABAQUS was established to predict the process-induced deformation of composite parts. The entire simulation calculation process is shown in Figure 3. In the model calculation, the heat transfer process of the composite parts is first simulated by the thermo-chemical model, and the temperature, cure degree and cure rate of each node during curing are calculated. Then, these calculated results are taken as known conditions in the thermo-mechanical model, and the residual stress during curing and the deformation after curing are investigated. Several useful user subroutine are compiled on FORTRAN language. In the thermo-chemical model, a user subroutine UMATHT is written to define the heat transfer model parameters of the composite and the cure kinetic equation of the resin based on Equations (1)–(3). In the thermo-mechanical model, a user subroutine UMAT is used to update the composite mechanical properties using Equation (8), and describe the Jacobin matrix and constitutive model based on Equation (6). Moreover, a user subroutine UEXPAN is written to describe the change of the non-mechanical strain generated by thermal contraction and chemical shrinkage.

**Figure 4:**
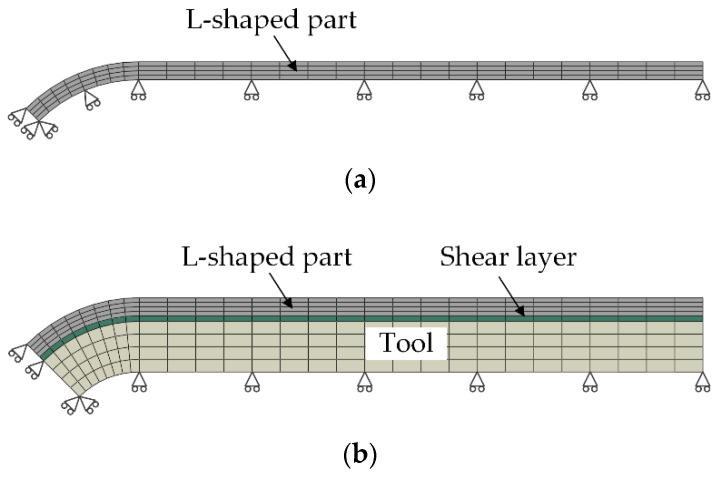
Finite element model of L-shaped parts: (**a**) without shear layer; (**b**) with shear layer.

In order to evaluate the influence of tool-part interaction on the deformation of composite parts, two finite element models with or without shear layer are established as shown in Figure 4. Considering symmetry, one-fourth of the composite part is modeled with 3-D solid elements. The eight-node linear heat transfer hexahedron element DC3D8 is selected in the thermo-chemical model, and the eight-node linear hexahedron element C3D8R is used in the thermo-mechanical model. One layer of the part in the thickness direction is modelled with one element. The shear layer and the tool in the thickness direction are modeled with one element and four elements, respectively. The element sizes of the corner and the flat flange of the part in the L-shaped parts are set as 1 mm × 1 mm × 0.16 mm and 2 mm × 1 mm × 0.16 mm, respectively. The mesh convergence analysis has been performed. When the mesh density of the part is doubled, the deformation change of the part is less than 1%. On the symmetric plane, the symmetry boundary conditions are used. During curing, the nodal displacements at the bottom surface of the tool or the part are constrained restrained normal to the surface. At the end of curing, the tool and shear layer are removed and all boundary conditions are released except the symmetry boundary conditions, the composite part is free to deform under residual stress. The finite element mesh and boundary conditions of the U-shaped parts are the same as those of L-shaped parts.

## 4. Results and Discussion

### 4.1. Model Validation

**Figure 5:**
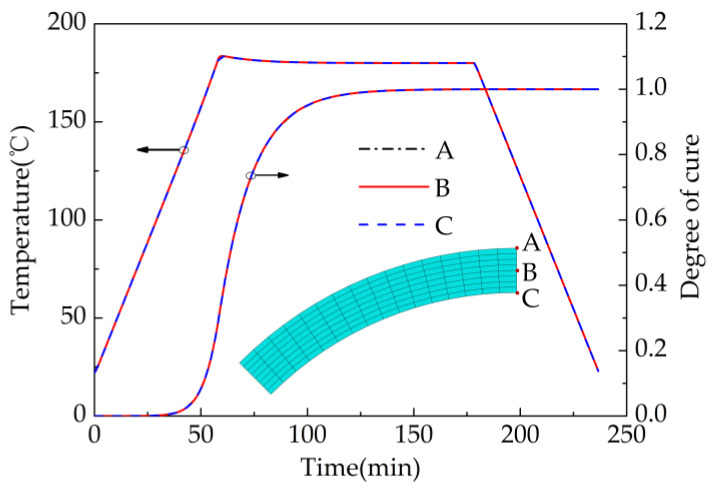
Predicted development of through-thickness temperature and cure degree for an eight-layer part during curing.

Figure 5 shows the predicted development of through-thickness temperature and cure degree for an eight-layer part during curing. The points A, B and C represent the top, center and bottom points in the thickness direction of the part, respectively. The predicted results show that the temperature and cure degree of the three points are nearly identical, and neither temperature gradient nor cure degree gradient was observed. The smaller thickness is the main reason for the absence of the temperature gradient and the cure degree gradient. The number of layers of all parts investigated in this paper is no more than eight. Therefore, it can be concluded that the influence of temperature gradient and cure degree gradient on deformation is very small and can be ignored in this paper.

**Figure 6:**
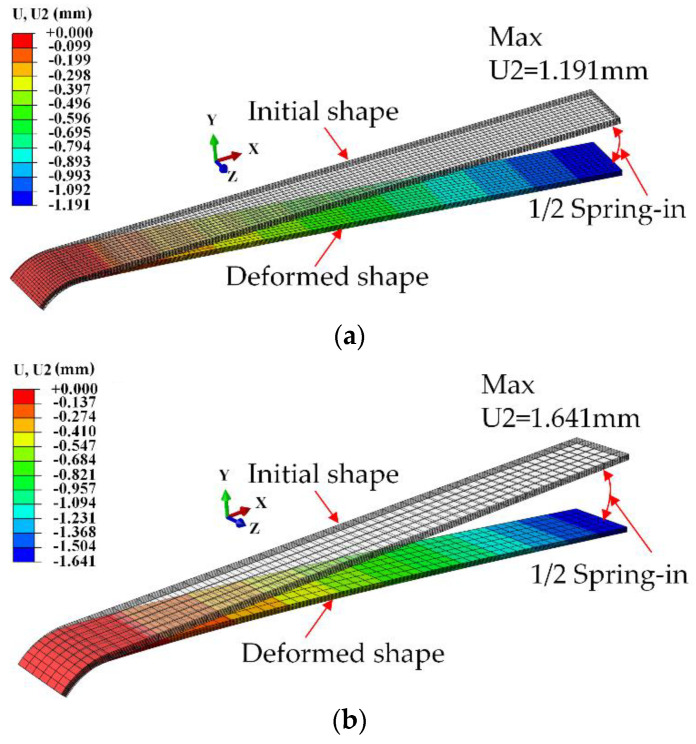
Predicted displacement nephogram and spring-in angle for a L-shaped part with [0/90_2_]_s_ lay-up: (**a**) without shear layer; (**b**) with shear layer. (The deformation scale factor is 8).

The deformation of a L-shaped part with [0/90_2_]_s_ lay-up is investigated, and the predicted displacement nephogram and spring-in angle of deformation are shown in Figure 6. It can be seen that the maximum Y-displacement of the part with shear layer is greater than that without shear layer, increasing by approximately 37.8%. The spring-in angles of L-shaped part with [0/90_2_]_s_ lay-up with and without shear layer are calculated as 1.788° and 1.285°, respectively. This proves that the tool-part interaction can significantly affect the deformation of composite parts and cannot be ignored.

**Figure 7:**
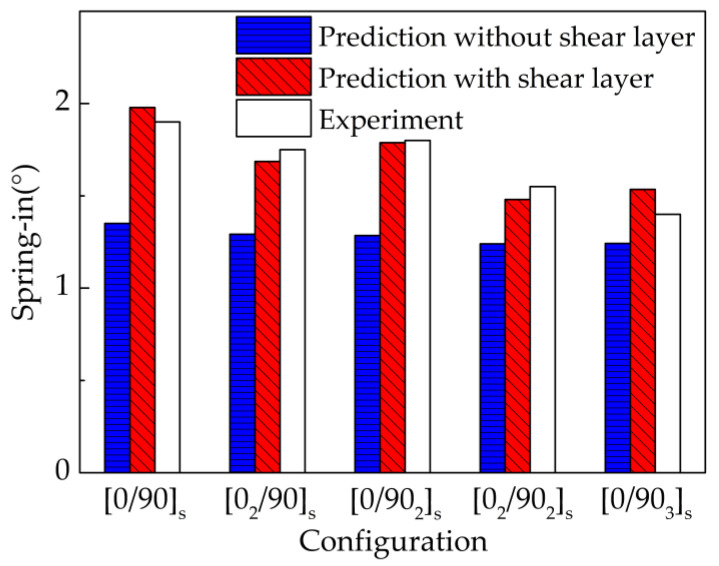
Comparison of the predicted and experimental spring-in of L-shaped parts.

Figure 7 shows the comparison of predicted and experimental spring-in of L-shaped parts. It can be observed that a large error exists between the predicted and the experimental results without shear layer, but the predicted results with shear layer are close to the experimental results, especially for thinner parts. A maximum relative error between the predicted results with shear layer and the experimental results is 9.64%. A good agreement between the predicted and experimental results proves that the proposed numerical model has high prediction accuracy.

**Figure 8:**
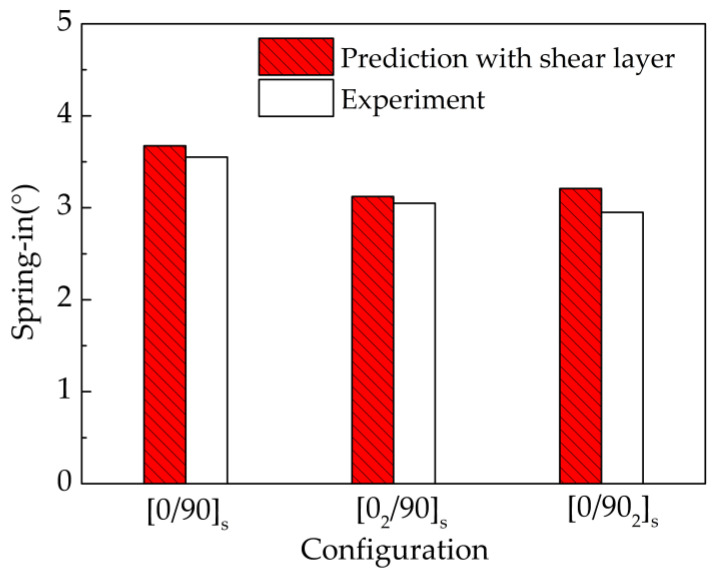
Comparison of the predicted and experimental spring-in for the U-shaped parts.

The rationality of the proposed numerical model is further verified by U-shaped parts. Figure 8 shows the comparison of predicted and experimental spring-in for U-shaped parts. It can be seen the differences between the predicted and experimental results are small, with the maximum relative error between them being 8.81%. Therefore, it can be concluded that the proposed numerical model has good generality and is suitable for predicting the deformation of L- and U-shaped parts.

Table 6 compares the prediction results of the proposed numerical model with those of previous literatures, and the predictive ability of different numerical models is revealed. In the literature [34], the maximum error of the prediction results of L-shaped parts is 10.8%, but the maximum error of U-shaped parts reaches 17.1%. However, the maximum error of the prediction results of L- and U-shaped parts from the proposed numerical model is less than 10%. These analysis results prove again that the proposed numerical model has a high prediction accuracy.

### 4.2. Quantifying the Influence of Structural Parameters on Spring-In

It can be seen from the above analyses that the thermal contraction, chemical shrinkage and tool-part interaction are the main spring sources of composite parts. In the following analysis, the proposed numerical model is used to investigate the relationship between the spring-in angles, three spring-in sources and structural parameters. Some interesting conclusions are obtained, which may help composite researchers better understand the mechanism of composite deformation.

**Figure 9:**
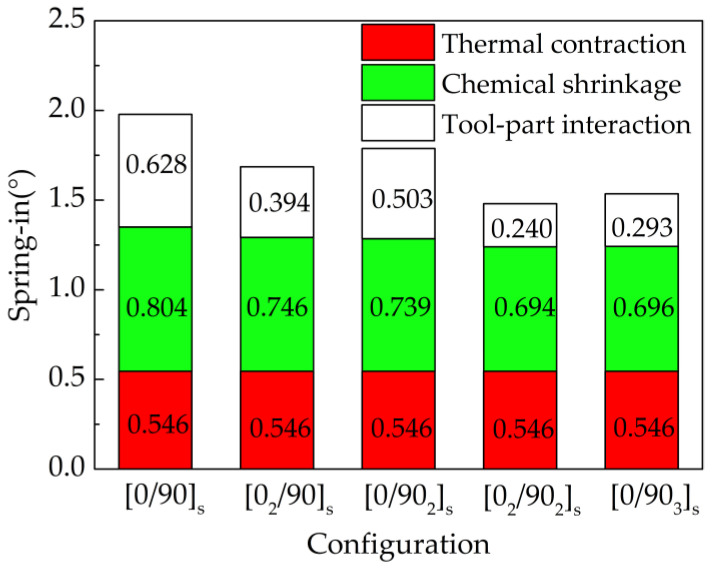
Spring-in caused by three spring-in sources for L-shaped parts.

Figure 9 shows the spring-in caused by three spring-in sources for L-shaped parts with different thicknesses and lay-ups. The spring-in angles caused by the thermal contraction, chemical shrinkage and tool-part interaction are denoted as ∆*θ*_ther_, ∆*θ*_chem_ and ∆*θ*_tool_, respectively. The following conclusions can be drawn from the figure:

(1) The ∆θther is always constant, and it changes slightly with the part thickness and lay-up.
(2) The ∆θchem gradually decreases with the increase of part thickness, while the lay-up has little influence on it.
(3) The ∆θtool gradually decreases with the increase of part thickness, and also decreases with the increase of bending stiffness due to the lay-up.

It is known from the above results that as the part thickness increases, both the spring-in angles caused by the chemical shrinkage and the tool-part interaction will decrease. The variation tendency of spring-in angle is the same, but the mechanisms are different. The increase of part thickness improves the bending rigidity of parts, which is the main reason for the reduction of spring-in angle caused by tool-part interaction. This has been described comprehensively in literature [25,27]. The phenomenon that the spring-in angle caused by chemical shrinkage decreases with increasing part thickness is caused by the shear-lag effect.

**Figure 10:**
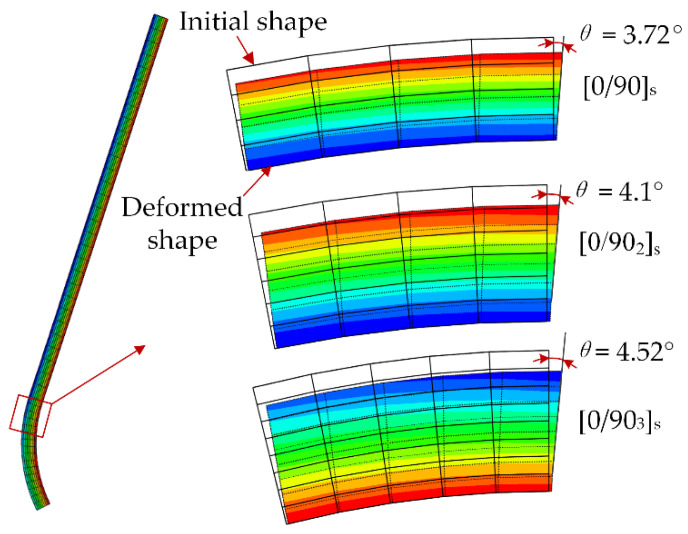
Predicted displacement nephogram of corner cross-section shape of L-shaped parts at the end of the rubbery stage. (The deformation scale factor is 20).

The effect of shear-lag on the spring-in of composite parts was quantitatively analyzed by the proposed numerical model. The chemical shrinkage occurs in the rubbery stage when the interlinear shear modulus of the composite is small. The shrinkage strain in the thickness direction causes slip deformation of the part cross-section shape, which results in the angle change in the normal direction of thickness. Figure 10 shows the deformation of the numerically simulated corner cross-section shape of the L-shaped part at the end of the rubbery stage. The results show that the corner cross-section deformation angles of [0/90]_s_, [0/90_2_]_s_ and [0/90_3_]_s_ are 3.72°, 4.1° and 4.52° with the deformation scale factor 20, respectively. It can be concluded that a thicker part leads to more severe shear-lag effect. The effect of shear-lag on spring-in can be simply explained from an energetic standpoint. When the chemical shrinkage strain energy is used to change the corner cross-section shape, the remaining strain energy for spring-in will be reduced. Therefore, with the increase of part thickness, the shear-lag effect in rubbery stage will increase, but the spring-in angle after curing will decrease.

**Figure 11:**
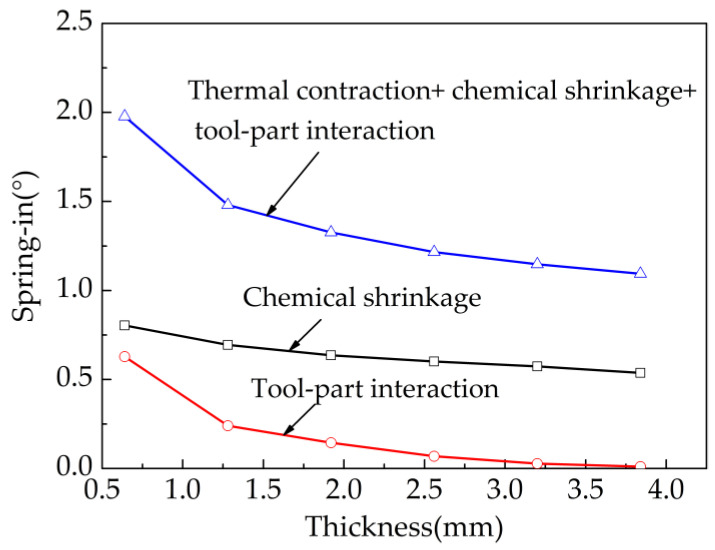
Influence of part thickness on spring for L-shaped parts.

The effect of part thickness on the spring-in angle of the part was further investigated. The basic form of the part lay-up is [0/90]_ns_ (n = 1,2,3…). Figure 11 shows the trend of the influence of part thickness on spring-in. The results suggest that:

(1) When the part thickness changes from 0.64 mm to 1.28 mm, the spring-in angle caused by chemical shrinkage decreases from 0.804° to 0.694° with a reduction percentage of 13.68%, and the spring-in angle caused by tool-part interaction decreases from 0.628° to 0.24° with a reduction percentage of 61.78%. It can be concluded that with the increase of thickness, the reduction rate of the spring-in angle caused by tool-part interaction is larger than that caused by chemical shrinkage.
(2) When the part thickness is 0.64 mm, 1.28 mm and 2.56 mm respectively, the proportional coefficients of spring-in angle caused by tool-part interaction are 31.78%, 16.22% and 5.67%, respectively. When the thickness exceeds 3.5 mm, the proportional coefficient reaches almost zero. It can be concluded that part thickness has a great influence on the spring-in angle caused by tool-part interaction.

## 5. Conclusions

In this paper, a 3-D numerical model considering the effect of tool-part interaction is proposed to predict the process-induced deformation of composite parts. The improved path-dependent model is applied to calculate the residual stress generated during curing, including the residual stress caused by the tool-part interaction in the viscous stage. The simplified self-consistent micromechanics model is used to predict composite mechanical properties in the viscous and rubbery stages. The numerically predicted results of L- and U-shaped parts with different thicknesses and lay-ups are compared with the experimental results. A good agreement proves that the proposed model has high prediction accuracy and good generality.

The influence of structural parameters on spring-in of composite parts is quantitatively analyzed. Some useful conclusions are summarized as follows: (1) the spring-in angles generated by thermal contraction are almost constant, and they change slightly with the part thickness and lay-up; (2) the spring-in angles caused by the tool-part interaction and chemical shrinkage decrease with the increase of part thickness; (3) with the increase of thickness, the shear-lag effect is enhanced, and more chemical shrinkage strain energy is consumed to change the cross-section shape of parts in the rubbery stage, and the spring-in caused by chemical shrinkage consequently becomes smaller after curing; (4) the reduction rate of spring-in caused by tool-part interaction is larger than that caused by chemical shrinkage. When the thickness exceeds 3.5 mm, the proportional coefficients of spring-in angle caused by tool-part interaction reach almost zero.

## Figures and Tables

**Table 1:** Thermal properties of T800HB/3900-2 composite.

Constant	Value
Composite density *ρ* (kg·m^−3^)	1600
Resin density *ρ_r_* (kg·m^−3^)	1380
Specific heat capacity coefficient *C* (J·kg^−1^·K^−1^)	925
Longitudinal thermal conductivity coefficient *k_x_* (W·m^−1^·K^−1^)	7.61
Transverse thermal conductivity coefficient *k_y_* = *k_z_* (W·m^−1^·K^−1^)	0.90
Total amount of heat *H_r_* (KJ·kg^−1^)	246

**Table 2:** Cure kinetic constants of 3900-2 resin [32].

Constant	Value
Pre-exponential coefficient *A* (min^−1^)	1.16 × 10^7^
Activation energy Δ*E* (J·mol^−1^)	7.25 × 10^4^
Exponential constant *m*	0.1781
Exponential constant *n*	1.2323

**Table 3:** Thermoelastic properties of T800HB/3900-2 composite in the glassy state [34].

Properties	Value
Longitudinal elastic modulus *E*_11_ (GPa)	169.0
Transverse elastic modulus *E*_22_, *E*_33_ (GPa)	8.62
In-plane shear modulus *G*_12_, *G*_13_ (GPa)	5.0
Transverse shear modulus *G*_23_ (GPa)	1.22
In-plane Poisson’s ratio *v*_12_, *v*_13_	0.355
Transverse Poisson’s ratio *v*_23_	0.410
Longitudinal CTE *β*_1_ (µε·°C^−1^)	−0.001
Transverse CTE *β*_2_, *β*_3_ (µε·°C^−1^)	29.5

CTE, coefficient of thermal expansion.

**Table 4:** Thermoelastic properties of aluminum and shear layer [25].

Properties	Aluminum	Shear Layer
Shear modulus (MPa)	26 × 10^3^	0.02
Poisson’s ratio	0.33	0.33
CTE (µε·°C^−1^)	23.6	23.6

**Table 5:** Thermoelastic properties of 3900-2 resin.

Properties	Values	Resource
Elastic modulus *E_r_* (MPa)	4710 (glassy)	[25]
	47.1 (rubbery)	[14]
	0.0471 (viscous)	[25]
Chemical shrinkage ∆*V_sh_*	0.03	[34]

**Table 6:** Comparison of model prediction accuracy.

Lay-up		Experiment (°) [34]	Prediction (°) [34]	Error (%)	Prediction of Proposed Model (°)	Error (%)
L-shaped	[0/90]_s_	1.9	2.07	9.1	1.978	4.11
	[0_2_/90]_s_	1.75	1.63	−7.0	1.686	−3.66
	[0/90_2_]_s_	1.8	1.61	−10.8	1.788	−0.67
	[0_2_/90_2_]_s_	1.55	1.42	−8.1	1.480	−4.52
	[0/90_3_]_s_	1.4	1.43	1.8	1.535	9.64
U-shaped	[0/90]_s_	3.55	4.16	17.1	3.674	3.49
	[0_2_/90]_s_	3.05	3.24	6.2	3.122	2.36
	[0/90_2_]_s_	2.95	3.21	8.7	3.210	8.81
